# Spilled gallstones found incidentally in a direct inguinal hernia sac: Report of a case

**DOI:** 10.1016/j.ijscr.2019.12.018

**Published:** 2019-12-19

**Authors:** Haci Bolat, Zafer Teke

**Affiliations:** a Nigde Omer Halisdemir University, Faculty of Medicine, Department of General Surgery, Nigde, Turkey; b Cukurova University, Faculty of Medicine, Department of Surgical Oncology, Adana, Turkey

**Keywords:** Laparoscopic cholecystectomy, Cholelithiasis, Complications, Spilled gallstones, Inguinal hernia, Hernia sac

## Abstract

**Introduction:**

Laparoscopic cholecystectomy (LC) is the preferred surgical treatment for symptomatic gallstones. Iatrogenic gallbladder perforation and spillage of gallstones during LC is a frequent occurrence. There are many different clinical presentations of complications resulting from dropped gallstones. We herein present a case of scattered gallstones after LC encountered incidentally during a direct inguinal hernia repair.

**Presentation of case:**

A 62-year-old male presented with a 4-year history of swelling of both right and left groins. He had undergone LC for acute calculous cholecystitis at another hospital 5 months earlier. Physical examination revealed reducible both right and left direct inguinal hernias. Surgical exploration of the right side revealed foreign bodies at the fundus of the sac attached to the inner wall, with a fibrotic reaction around it. On closer inspection these foreign bodies were macroscopically consistent with gallstones. The gallstones were removed, and bilateral herniotomies and Lichtenstein's prolene mesh repair were performed. Pathologic evaluation confirmed 10 foreign bodies of 5-mm in size to be cholesterol gallstones.

**Discussion:**

Gallstones have been very rarely reported previously within a hernia sac after LC. Most of the spilled gallstones are clinically silent and rarely become symptomatic. Complications may occur from the immediately postoperative period to a long time interval of 20 years. Treatment of complications is based on its type and location.

**Conclusion:**

This case presents a very rare entity resulting from leaving spilled gallstones behind. We recommend that every effort should be made to retrieve any scattered stones during LC in order to avoid complications.

## Highlights

• Iatrogenic gallbladder perforation and spillage of gallstones during laparoscopic cholecystectomy is a frequent occurrence.
• Most of the spilled gallstones are clinically silent and rarely become symptomatic.
• Complications may occur from the immediately postoperative period to a long time interval of 20 years.
• Gallstones have been very rarely reported previously within a hernia sac after laparoscopic cholecystectomy.
• It is recommended that every effort should be made to remove any scattered gallstones during laparoscopic cholecystectomy.

## 1 Introduction

Laparoscopic cholecystectomy (LC) is the preferred surgical treatment for symptomatic gallstones. The laparoscopic procedure is superior to the open approach in many aspects. However, iatrogenic gallbladder perforation and spillage of gallstones during LC is a frequent occurrence with rates reported between 1.4 % and 40 % [1]. There are many different clinical presentations of complications resulting from dropped gallstones during LC.

In this article, we aimed to present a case of scattered gallstones after LC encountered incidentally during a direct inguinal hernia repair. This work is reported in line with the Surgical CAse REport (SCARE) Guidelines criteria [2].

## 2 Case presentation

A 62-year-old male presented with a 4-year history of swelling of both right and left groins which had been causing increasing discomfort over the previous 3 months. He had undergone LC for acute calculous cholecystitis at another hospital 5 months earlier. As far as we learned, the procedure had been unremarkable except for spillage of bile and gallstones into the peritoneum. Most of the stones had been retrieved during surgery.

**Fig. 1:**
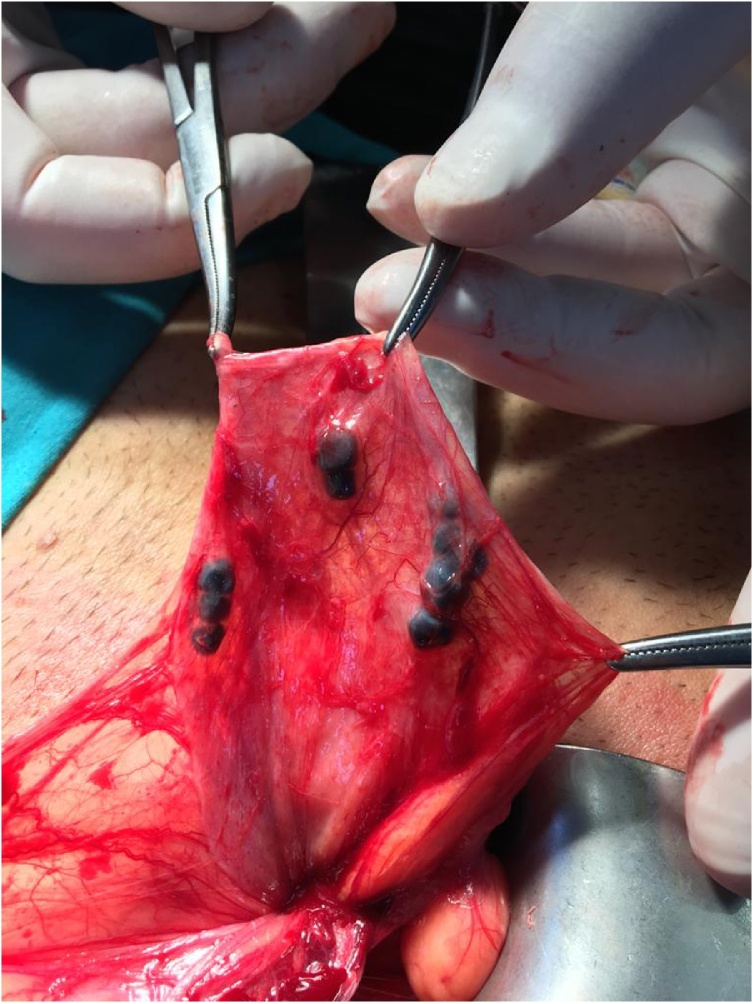
Gallstones at the fundus of the hernia sac attached to the inner wall.

**Fig. 2:**
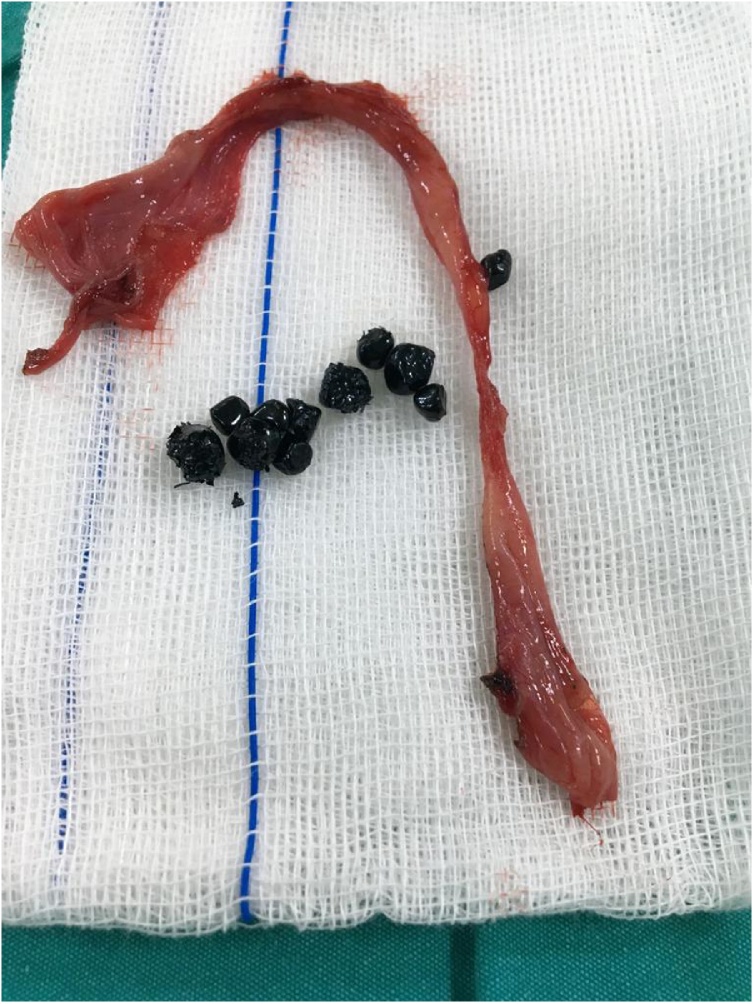
Excised right inguinal hernia sac with ten 5 mm gallstones.

Physical examination revealed reducible both right and left direct inguinal hernias. Abdominal and testicular examinations were otherwise unremarkable. Diagnosis was bilateral inguinal hernia. Surgical exploration of the right side revealed foreign bodies at the fundus of the sac attached to the inner wall, with a fibrotic reaction around it (Fig. 1). On closer inspection these foreign bodies were macroscopically consistent with gallstones. The gallstones were removed, and bilateral herniotomies and Lichtenstein's prolene mesh repair were performed. The hernia sac and foreign bodies were sent to the pathologist (Fig. 2). Pathologic evaluation confirmed 10 foreign bodies of 5-mm in size to be cholesterol gallstones. The patient was discharged home two days later.

## 3 Discussion

LC is a procedure associated with low morbidity and mortality. The incidence of gallbladder perforations with spilled bile or gallstones has been found to be as high as 40 % (0.1 %–40 %), although complications resulting from retained gallstones are rare, occurring with estimated rates between 0.08 % and 8.5 % [3]. Important risk factors that increase the risk of gallbladder perforation and gallstone spillage are lower experience of surgeons with laparoscopic procedure, male sex, older age, and acute cholecystitis [4].

During laparoscopic surgery, the gallbladder may be torn by the penetrating bites of the grasping instrument or sheared by back and forth traction on the gallbladder wall as it is moved to enhance exposure. The gallbladder may be inadvertently entered by using monopolar electrosurgical hook during its dissection from the liver bed. The risk of perforating the gallbladder when it is subjected to physical strain becomes greater under conditions of acute inflammation. In acute cholecystitis, gallbladder wall is edematous and fragile, and the presence of omental adhesions or phlegmon makes dissection difficult, which may result in tearing of the wall. Finally, stone spillage may occur during the forced delivery of a freed tense gallbladder through the small port.

Most of the spilled gallstones are clinically silent and rarely become symptomatic. At presentation, they almost have unfamiliar clinical patterns in unusual locations. Besides, because of a long delay between the first operation and incidence of complications, the diagnosis is usually challenging. They pose a diagnostic challenge in accidental radiologic imagings, because they present as hypodense to hyperdense nodules mimicking peritoneal seeding. This problem becomes prominent particularly in patients with a history of malignancy of other organs. Computed tomography scan and ultrasound imaging are advocated as the best options for the diagnosis of complications caused by spilled gallstones [5].

The incidence of early and late complications of lost gallstones is actually low. Complications may occur from the immediately postoperative period to a long time interval of 20 years. Gallbladder perforation can cause more early postoperative complications such as pain, fever, ileus, and trocar site infection. Prevalent complications of lost gallstones are intraabdominal abscesses with or without discharging abdominal wall fistula tract, abdominal wall sinus tracts or collections of trocar sites, bilio-colono-cutaneous fistula, peritoneal microabscesses and granuloma, liver abscess, retroperitoneal abscess, and rarely thoracic complications even after a long time interval [1]. The most common complications arise from the peritoneal cavity, which may present in the form of intraabdominal abscesses with or without transabdominal fistulas, with an estimated incidence of 0.3%–2.9% [6]. Other uncommon presentations of lost gallstones are urinary tract involvement, obstructive cholangitis, incarceration in the hernia sac, acute appendicitis, middle colic vessel thrombosis, intestinal volvulus, and obstruction. Gallstones have been extremely rarely reported previously within a hernia sac after LC [7,8]. Treatment of complications is based on its type and location.

It is widely accepted that every effort should be made to remove any scattered stones during LC. This could be accomplished by several methods. In the event of a gallbladder perforation, closure of the hole should be attempted with laparoscopic graspers, hemoclips, endoloops, or a laparoscopic ligature. Surgeons should attempt to collect spilled bile by copious irrigation of peritoneal cavity and locate them with oblique view scopes or using additional working cannulas, and should retrieve as many stones as possible laparoscopically. Special care should be taken not to disperse stones through the peritoneal cavity when using irrigation. Extension of the port site incision in order to extract the gallbladder intact is a good choice short of risking spillage of gallstones when trying to force the gallbladder through an undersized port site. As a preventive measure to stone loss during extraction, endobags are normally used to contain the gallbladder and possible loose stones. Lost stones do not warrant a conversion to open surgery [9]. If gallstones cannot be retrieved, it is recommended to record lost gallstones in operative notes and inform the patients about possible complications, and to follow them up closely.

## 4 Conclusion

Gallstones have been very rarely reported previously within a paraumblical incisional hernia sac, an umbilical trocar site hernia sac and several inguinal hernia sacs after LC [1,[7], [8], [9]]. The patient described had gallstones spilled intraperitoneally during gallbladder dissection from the liver bed. The gallstones then migrated transcoelomically downward to settle in a right inguinal hernia sac. Later, the stones induced an inflammatory response resulting in fibrous tissue at the hernial fundus. This case presents a very rare entity resulting from leaving spilled gallstones behind. We recommend that every effort should be made to retrieve these stones in order to avoid complications.

## Sources of funding

This research did not receive any specific grant from funding agencies in the public, commercial, or not-for-profit sectors.

## Ethical approval

I certify that this study does not require ethical approval.

## Consent

Written informed consent for publication of the patient’s clinical details and images was obtained from the patient.

## Author contribution

Haci Bolat and Zafer Teke - study concept and design, data collection, data analysis and interpretation, writing the paper, final decision to publish

## Registration of research studies

We registered our study with the Research Registry. Our unique identifying number is: researchregistry5173.

## Guarantor

Haci Bolat

## Provenance and peer review

Editorially reviewed, not externally peer-reviewed

## Declaration of Competing Interest

None.

## References

[1] Jabbari Nooghabi A., Hassanpour M., Jangjoo A. (2016). Consequences of lost gallstones during laparoscopic cholecystectomy: a review article. Surg. Laparosc. Endosc. Percutan. Tech..

[2] Agha R.A., Borrelli M.R., Farwana R., Koshy K., Fowler A.J., Orgill D.P., SCARE Group (2018). The SCARE 2018 statement: Updating consensus Surgical CAse REport (SCARE) guidelines. Int. J. Surg..

[3] Sathesh-Kumar T., Saklani A.P., Vinayagam R., Blackett R.L. (2004). Spilled gallstones during laparoscopic cholecystectomy: a review of the literature. Postgrad. Med. J..

[4] Brockmann J.G., Kocher T., Senninger N.J., Schürmann G.M. (2002). Complications due to gallstones lost during laparoscopic cholecystectomy. Surg. Endosc..

[5] Nayak L., Menias C.O., Gayer G. (2013). Dropped gallstones: spectrum of imaging findings, complications and diagnostic pitfalls. Br. J. Radiol..

[6] Rice D.C., Memon M.A., Jamison R.L., Agnessi T., Ilstrup D., Bannon M.B., Farnell M.B., Grant C.S., Sarr M.G., Thompson G.B., van Heerden J.A., Zietlow S.P., Donohue J.H. (1997). Long-term consequences of intraoperative spillage of bile and gallstones during laparoscopic cholecystectomy. J. Gastrointest. Surg..

[7] Rosin D., Korianski Y., Yudich A., Ayalon A. (1995). Lost gallstones found in a hernial sac. J. Laparoendosc. Surg..

[8] Aspelund G., Halldorsdottir A.B., Isaksson H.J., Moller P.H. (1999). Case of the month. Gallstone in hernial sack. Laeknabladid.

[9] Demirbas B.T., Gulluoglu B.M., Aktan A.O. (2015). Retained abdominal gallstones after laparoscopic cholecystectomy: a systematic review. Surg. Laparosc. Endosc. Percutan. Tech..

